# Biosynthesis and Characterization of Recombinant Silk-Like Polypeptides Derived from the Heavy Chain of Silk Fibrion

**DOI:** 10.3390/polym9120669

**Published:** 2017-12-03

**Authors:** Yue Wu, Zhao Kang, Zhifang Tian, Mingyang Wu, Jiannan Wang

**Affiliations:** National Engineering Laboratory for Modern Silk, College of Textile and Clothing Engineering, Soochow University, No. 199 Ren-ai Road, Suzhou Industrial Park, Suzhou 215123, Jiangsu Province, China; 20154215026@stu.suda.edu.cn (Y.W.); 20165215005@stu.suda.edu.cn (Z.K.); tianzhifang1989@163.com (Z.T.); 20144215066@stu.suda.edu.cn (M.W.)

**Keywords:** *Bombyx mori*, silk fibroin, heavy chain, protein expression, *E. coli*, characterization

## Abstract

In order to investigate the impacts on the structure and biomedical function of typical fragments derived from repetitive and non-repetitive regions of the *Bombyx mori* silk fibroin heavy chain, several block combination genes (*gs16f1, gs16f4, gs16f8, and gs16f12*) were designed, cloned into a fusion protein expression vector tagged with glutathione S-transferase (GST), and expressed in *Escherichia coli*. Fusion proteins GST-GS16F1, GST-GS16F4, and GST-GS16F8 were purified by GST affinity chromatography, and single bands were identified by SDS-PAGE. Under optimal initial cell density, in ducer concentration and induction expression time, the yield of purified GST-GS16F1, GST-GS16F4, and GST-GS16F8 per liter of bacterial culture reached 79, 53, and 28 mg, respectively. Mass spectrometry revealed molecular weights for GST-GS16F1, GST-GS16F4, and GST-GS16F8 of 37.7, 50.0, and 65.7 kDa, respectively, consistent with the theoretical values of 37.4, 49.4, and 65.5 kDa. Similarly, measured values of pI were 5.35, 4.5, and 4.2 for the fusion proteins, consistent with predicted values of 5.34, 4.44, and 4.09. CD spectra showed the molecular conformation of GS16F1 was mainly *β*-sheet structure, while more stable *α*-helix structure formed in GS16F4 and GS16F8.

## 1. Introduction

*Bombyx mori* (*B. mori*, Lepidoptera) produces silk fibroin that is linked to biomedical applications owing to its high biocompatibility, degradability and ease of fabrication [1,2,3,4,5,6]. Silk fibroin from *B. mori* comprises a fibroin heavy chain (H-chain), a light chain (L-chain), and a glycoprotein (P25), and complete gene and amino acid sequences have been reported. H-Chain is the major component and includes an N-terminal region, a major core region, and a C-terminal region. The core domain consists of 12 repetitive domains and 11 non-repetitive fragments [7], and this portion can be used as a model structure to investigate the relationship between structure and function, including cell functions. 

Silk fibroin is a crystalline polymer composed of three crystalline modifications, silkI, silkII, and silkIII [8,9,10,11]. Numerous reports have discussed the relationship between the sequence, conformation and characteristics of silk fibroin analogues based on chemical synthesis and genetic engineering methods. The (GAGAGS) hexapeptide is the core unit of H-Chain and plays an important role in the formation of crystalline domains [12,13]. Silk protein-like multiblock polymers derived from the repetitive domain of *B. mori* silk and spider dragline silk spontaneously aggregate into β-sheet structures, similar to natural silks [14,15,16]. A model polypeptide (Ala-Gly)*n* (*n* = 12 and 5–9) has been synthesized and its structure reported, and the structure affects the degree of polymerization [17]. Prokaryotic expression systems based on *E. coli* are regularly used to express heterologous proteins with high molecular weights [18]. A block copolymer containing elastin (GVGVP) and silk (GAGAGS) motifs has been biosynthesized and analyzed [19]. A full-length recombinant protein derived from hornet has also been produced by *E. coli* and displays high cell adhesion activity [20]. Markus et al. [21] expressed recombinant spider flagelliform silk domains and investigated the effects of the various domains on assembly and solubility. A type of silk-elastin-like polymer, composed of repetitive silk fibroin (GAGAGS) and mammalian elastin (VPGVG) motifs, was expressed in *E. coli* and enhanced cell viability slightly [22]. A recombinant spider silk protein with the cell-binding Arg-Gly-Asp (RGD) motif has been produced in *E. coli* and its cell adherence properties were explored [23].

In our previous work, repetitive GAGAGX polypeptides (X is A, Y, V, or S) derived from the H-Chain were designed and produced using chemical synthesis [15]. These four polypeptides aggregate into different molecular conformations by self-assembly. We also biosynthesized all four (GAGAGX)_16_ repetitive polypeptides via genetic engineering and explored their molecular conformations [24,25], and a similar study on non-repetitive regions of Fn (*n* = 1, 2, 3, 4, 8, and 12) has recently been completed [26,27]. The major aim of the present work was to produce recombinant proteins composed of the (GAGAGS)_16_ repetitive fragment and the non-repetitive F1, F4, and F8 fragments in a prokaryotic system and investigate the characteristics of the expression products.

## 2. Materials and Methods

### 2.1. Materials

**Figure 1:**
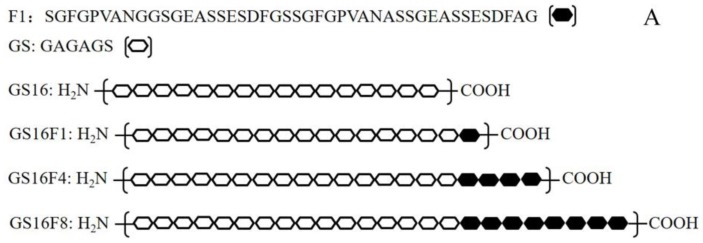

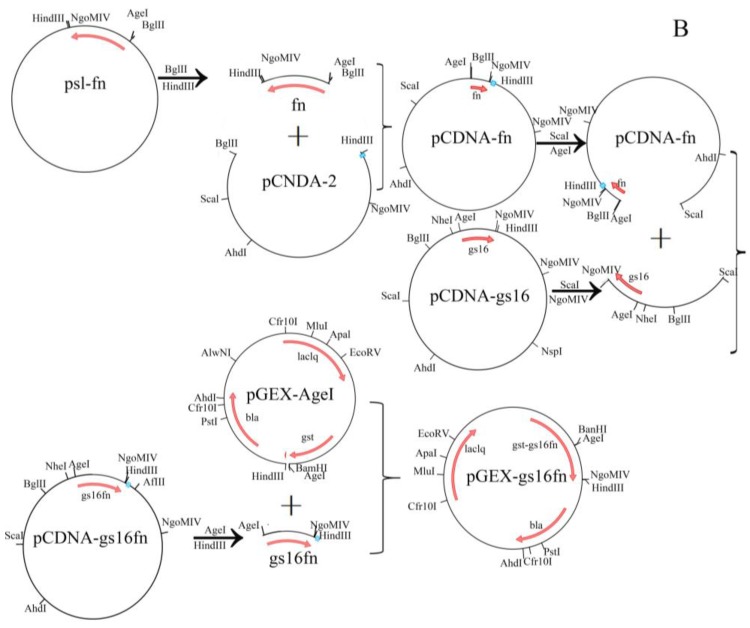
Designed amino acid sequences of the recombinant H-Chain (**A**) and a schematic diagram of the expression vector (**B**).

The expression vector pGEX-AgeI (modified pGEX-KG), and plasmids pCDNA-2, pCDNA-*gs16* carrying a *gs16* gene encoding the repetitive fragment of H-Chain (GS16, Figure 1A) [25], and pSL-*fn* (*n* = 1, 2, 3, 4, 8, and 12) [26] were constructed and stored at −80 °C in our laboratory, along with *E. coli* DH5*α* and the expression strain BL21 (DE3). The *fn* fragments are gene sequences encoding non-repetitive sequences of H-Chain and all multimers (F1, F4, and F8, Figure 1A). Restriction endonucleases *Bgl*II, *Hind*III, *Age*I, and *Bam*HI were purchased from Fermentas (vilnius, Lithuania), *NgoM*IV was purchased from Promega (Madison, WI, USA), and T4 DNA ligase, *Sca*I and DNA molecular weight standards were purchased from Takara (Kusatsu, Japan). Agarose for DNA electrophoresis was purchased from the Gene Company (Hong Kong, China).

### 2.2. Vector Construction 

Recombinant genes were cloned as previously described [28]. Gene fragment and all multimers were digested from pSL-*fn* with *Bgl*II and *Hind*III, then inserted into pCDNA-2 pre-digested with the same enzymes to obtain pCDNA-*fn*. Plasmid pCDNA-*gs16* was digested with *Sca*I and *NgoM*IV, and the resultant *gs16* fragment was inserted into pCDNA-*fn* pre-digested with *Age*I and *Sca*I to obtain pCDNA-*gs16fn*. Recombinant *gs16fn* genes were excised from pCDNA-*gs16fn* using *Age*I and *Hind*III and inserted into the expression vector pGEX-AgeI to generate GST gene fusions (Figure 1B). An *Age*I site was inserted in pGEX-KG to obtain plasmid pGEX-AgeI [25]. The resulting expression vectors were designated pGEX-*gs16f1*, pGEX-*gs16f2*, pGEX-*gs16f3*, pGEX-*gs16f4*, pGEX-*gs16f8*, and pGEX-*gs16f12*. All vectors were further verified by nucleotide sequencing (Invitrogen, Carlsbad, CA, USA).

### 2.3. Agarose Gel Electrophoresis

Agarose gel electrophoresis were performed as previously described [28]. Briefly, plasmids pCDNA-(*gx16 + f*) were digested with restriction enzymes *Bgl*II and *Hind*III, and expression vectors pGEX-(*gx16 + f*) were digested with restriction enzymes *Bam*HI and *Hind*III at 37 °C for 15 min. After digestion, electrophoresis was performed using a 1% agarose gel in 1 × TAE buffer (0.04 M Tris-acetic acid, 1 mM ethylene diamine tetraacetic acid (EDTA)) and visualized by ethidium bromide staining.

### 2.4. Protein Expression and Purification 

Fusion proteins were expressed and purified as previously described [25]. Briefly, expression vectors pGEX-*gs16f1*–pGEX-*gs16f12* were transformed into *E. coli* BL21 (DE3) cells and cultured on Luria–Bertani (LB) solid medium containing ampicillin at 37 °C for 12–16 h. A single colony was picked and inoculated into 4 mL LB medium containing ampicillin and cultured at 37 °C overnight, then inoculated into 250 mL of fresh LB medium and cultured. When cell destiny OD_600_ up to 0.6–0.8, IPTG was added to induce expression of the GST-GS16F1, GST-GS16F2, GST-GS16F3, GST-GS16F4, GST-GS16F8, or GST-GS16F12 fusion proteins. The initial cell densities (OD_600_ = 0.3–2.1 AU, in 0.3 AU intervals) and IPTG concentrations (0–1.0 mM, in 0.1 mM intervals) were tested to optimize protein expression. At different induction time points from 0 to 8 h, cells were harvested by centrifugation at 4 °C. The cell pellet was re-suspended in GST binding buffer and sonicated on ice. After centrifugation at 4 °C, loading the supernatant onto a GST affinity column (Novagen, Billerica, MA, USA), and eluting fusion protein with GST elution buffer containing 10 mM reduced glutathione.

### 2.5. SDS-PAGE 

Protein molecular weights were determined qualitatively by SDS-PAGE as described previously [25]. Briefly, loading buffer was added to whole cell lysates or purified fusion proteins and boiled for 3–5 min. Then samples were separated by SDS-PAGE with a 10% (*w/v*) polyacrylamide gel (Sigma, St. Louis, MO, USA) and stained by Coomassie brilliant blue.

### 2.6. Mass Spectrometry

Purified fusion proteins were desalted with a ZipTip C4 column (Millipore, Bellirica, MA, USA) and quantitative analysis was performed using a 4800 MALDI-TOF/TOF mass spectrometer (MS) (AB SCIEX, Foster City, CA, USA).

### 2.7. Determination of Expression Yield 

The yield of purified fusion protein was determined using a Smartspec Plus Ultraviolet spectrophotometer (Bio-Rad, Hercules, CA, USA) by measuring the absorbance at 260 and 280 nm. Protein concentration was calculated using the formula C (mg/mL) = 1.45 × *A*_280_ − 0.74 × *A*_260_, then converted to amount per L of bacterial cell culture.

### 2.8. Determining the Amino Acid Composition

Amino acid composition was determined as previously described [25]. Briefly, purified fusion proteins were diluted to 0.05 mg/mL and filtered to remove impurities. Peptide bonds were hydrolysed using 6 M HCl, and free amino acids derivatised using phenyl isothiocyanate (P1034-1ML, Sigma, St. Louis, MO, USA) and tested using LC-20A high-performance liquid chromatography (Shimadzu Corp., Kyoto, Japan).

### 2.9. Isoelectric Point Measurement 

Isoelectric points were measured as previously described [27]. Briefly, the *ζ*-potential of each sample was measured using a ZS90 Zetasizer Nano (Malvern Instruments, Malvern, UK) in sodium phosphate buffer at 25 °C. The pH of the buffer were adjusted to 2, 3, 4, 5, 6, or 7 using NaOH or HCl. Each sample was analyzed in triplicate.

### 2.10. Circular Dichroism (CD) Assay

Per milligram purified fusion protein was digested using 1 U thrombin (Novagen) in lysate buffer at 20 °C for 16 h, as previously described [25]. The diluted reaction mixture was loaded onto a GST-affinity column to remove the GST-tag and collect the polypeptides GS16F1, GS16F4, and GS16F8. Then the polypeptides were detected via a J-815 CD spectrometer (Jasco, Tokyo, Japan), using a 1.0 mm path-length cell at 25 °C with an accumulation time of 4 s and a scanning rate of 100 nm·min^−1^.

## 3. Results and Discussion

### 3.1. Characteristics of Vectors

**Figure 2:**
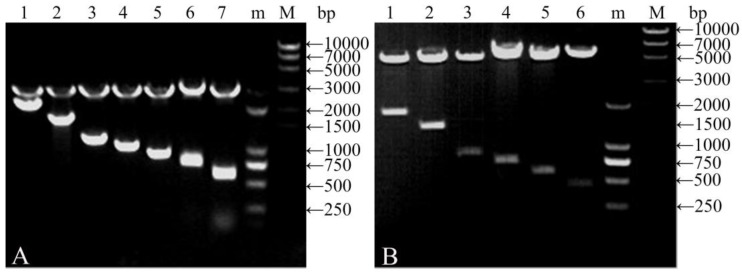
DNA gel electrophoretogram of clone plasmids digested with *Bgl*II/*Hind*III (**A**) and expression vectors digested with *Bam*HI/*Hind*III (**B**). (M and m): DNA molecular weight standards. (**A**) Lane 1, pCDNA-*gs16f12*; Lane 2, pCDNA-*gs16f8*; Lane 3, pCDNA-*gs16f*4; Lane 4, pCDNA-*gs16f3*; Lane 5, pCDNA-*gs16f2*; Lane 6, pCDNA-*gs16f1*; and Lane 7, pCDNA-*gs16*. (**B**) Lane 1, pGEX-*gs16f12*; Lane 2, pGEX-*gs16f8*; Lane 3, pGEX-*gs16f4*; Lane 4, pGEX-*gs16f3*; Lane 5, pGEX-*gs16f2*; and Lane 6, pGEX-*gs16f1*.

The plasmids pCDNA-*gs16f1–12* containing the repetitive fragment and non-repetitive gene fragments were confirmed by agarose gel electrophoresis following digestion with *Bgl*II and *Hind*III. Two bands were excised from each plasmid; a 2913 bp vector-derived fragment, and a fragment of varying size corresponding to the repetitive motif segment (Figure 2A). The sizes of the smaller bands corresponding to the repetitive fragments were of the expected sizes of 712, 844, 976, 1108, 1240, and 2164 bp. Plasmid pCDNA-*gs16* served as a control and produced 2913 and 587 bp fragments.

Expression vectors pGEX-*gs16f1–12* containing the recombinant repetitive and non-repetitive gene fragments were qualitatively confirmed by agarose gel electrophoresis following digestion with *Bam*HI and *Hind*III. Two bands were observed for each expression vector; a 4943 bp fragment corresponding to the vector, and a fragment of varying size corresponding to the repetitive or non-repetitive motif segment (Figure 2B). The sizes of the smaller fragments excised from pGEX-g*s16f1–12* were of the expected sizes of 432, 564, 696, 828, 1356, and 1884 bp.

### 3.2. Fusion Protein Expression and Optimisation

**Figure 3:**
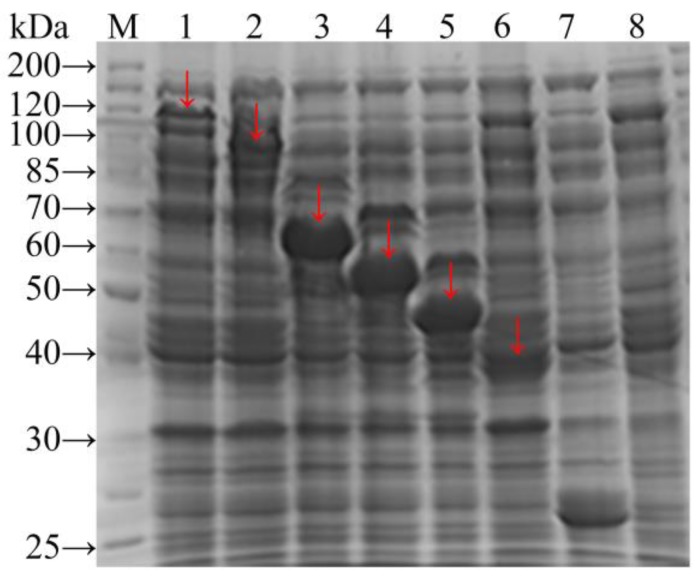
SDS-PAGE electrophoretogram of *E. coli* BL21 total proteins. Lane M: protein molecular weight standards. Lanes 1–7: containing the expression vector pGEX-*gs16f12*, pGEX-*gs16f8*, pGEX-*gs16f4*, pGEX-*gs16f3*, pGEX-*gs16f2*, pGEX-*gs16f1*, and pGEX-*AgeI*, respectively. Lane 8: not containing the expression vector.

As described previously for the expression of repetitive and non-repetitive fragments [25,26], recombinant genes were expressed under the regulation of the Ptac promoter, which includes a translation-enhancing sequence (*g10*), and a ribosome-binding site for transcriptional control. The fused GST tag was applied to purify the expression products, and was cleaved by thrombin. The ATG initiation codon was upstream of the GST gene, and the TGA termination codon followed the *gs16fn* target gene. Expression of *gs16fn* (*n* = 1, 2, 3, 4, 8, and 12) was first evaluated in crude cell extracts by SDS-PAGE. Fusion proteins GST-GS16F12, GST-GS16F8, GST-GS16F4, GST-GS16F3, GST-GS16F2, and GST-GS16F1 all showed clear bands (Figure 3, lanes 1–6, respectively, indicated by red labels).

The theoretical molecular weights of GST and GST-GS16Fn (*n* = 1, 2, 3, 4, 8, and 12) are 26.1, 37.4, 41.4, 45.4, 49.4, 65.5, and 81.4 kDa, respectively. It can be seen from SDS-PAGE that the experimentally determined molecular weights of GST and GST-GS16F1 fusion protein were generally corresponding to the predicted values. However, the molecular weights of GST-GS16F2–GST-GS16F12 were larger than predicted, and deviated further from the molecular weight standards with an increasing number of F1 repeats. We, therefore, decided to confirm the molecular weights of purified fusion proteins using mass spectrometry.

**Figure 4:**
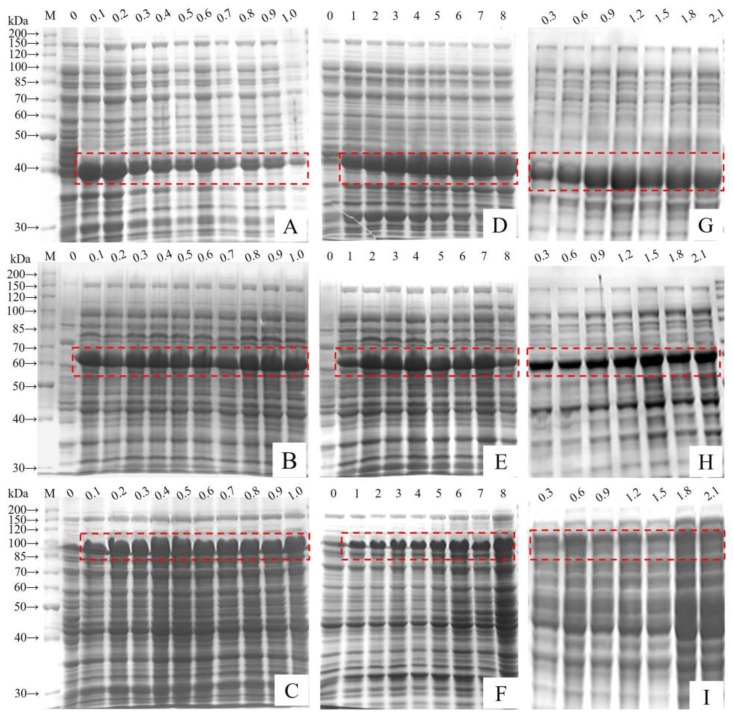
SDS-PAGE electrophoretogram of *E. coli* BL21 total proteins under different expression conditions. (**A**–**C**) different IPTG concentration (mM); (**E**,**F**) different induce time (h); (**G**–**I**): different initial cell density (OD_600_). (**A**,**D**,**G**): GST-GS16F1; (**B**,**E**,**H**): GST-GS16F4; and (**C**,**F**,**I**): GST-GS16F8.

In order to obtain sufficient quantities of the designed polypeptides to explore their structures and biological properties, expression levels of the fusion proteins were optimized by varying the IPTG concentration, the induction time, and the initial cell density. When the initial cell density, up to OD_600_ = 0.6 AU, protein expression was induced using IPTG between 0 and 1 mM, and culturing continued for 6 h. Expression was minimal without IPTG induction, bands were visible with 0.1 mM IPTG, and 0.2 mM IPTG was found to be optimal for GST-GS16F1 expression, while 0.1 mM was optimal for GST-GS16F4 expression and 0.4 mM was optimal for GSTGS16F8 expression (Figure 4A–C). Using the optimal IPTG concentration and cell density (OD_600_ = 0.6 AU), expression levels were evaluated over different culturing periods. The expression levels of all fusion protein variants were improved by increasing the induction time (Figure 4D–F). Following a 1 h induction, GST-GS16F1 expression level were already appreciable, and levels did not increase after a 3 h induction. GST-GS16F4 expression was increased significantly following a 2 h induction and peaked after 4 h. GST-GS16F8 expression peaked after a 6 h induction, with no subsequent change after 8 h. Using the optimal IPTG concentration and induction time, the optimal cell density was investigated, and optimal OD_600_ values were 1.2 AU for GST-GS16F1 expression, 1.5 AU for GST-GS16F4 expression, and 1.8 AU for GST-GS16F8 expression (Figure 4G–I).

### 3.3. Purification of Fusion Proteins and MS Analysis 

**Figure 5:**
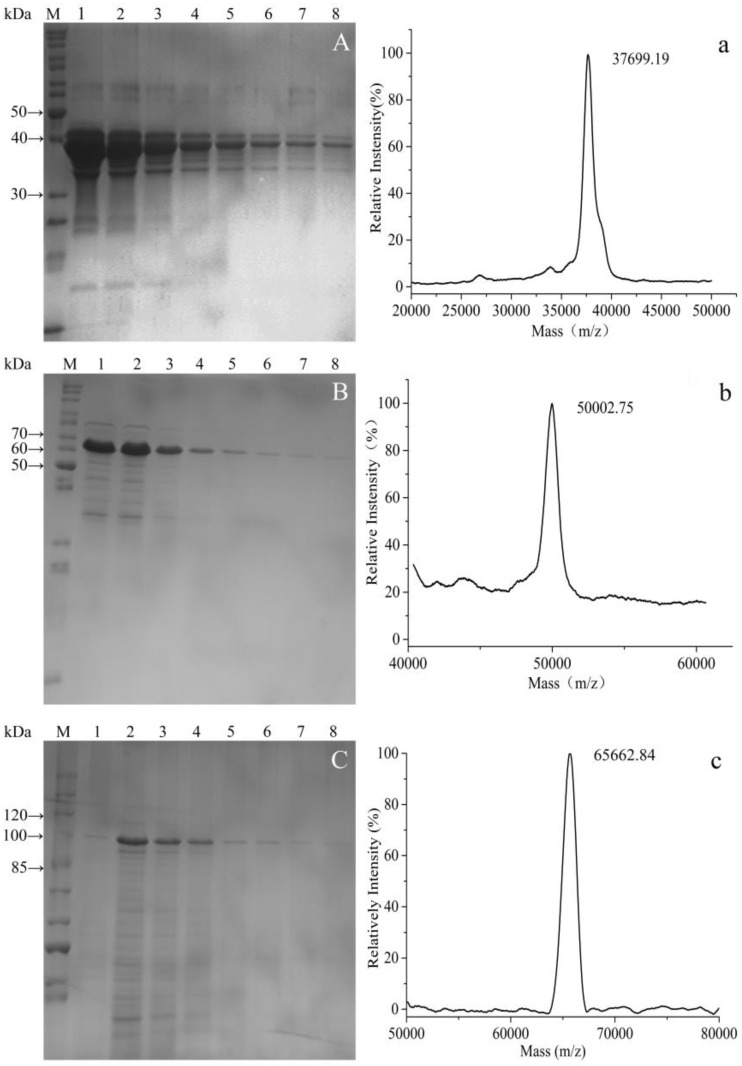
SDS-PAGE electrophoretogram (**A**–**C**) and MS spectrum (**a**–**c**) of purified fusion proteins. (**A**,**a**) GST-GS16F1; (**B**,**b**) GST-GS16F4; (**C**,**c**) GST-GS16F8. Lanes 1–8 were collected in successive tubes (1 mL/tube).

Protein purification was performed using the attached GST tags. SDS-PAGE analysis indicated that all fusion proteins were purified to high degree following affinity chromatography (Figure 5A–C). SDS-PAGE of the purified fusion proteins also revealed that the molecular weight of GST-GS16F1 was close to the theoretical value, while those of GST-GS16F4 and GST-GS16F8 were both larger than predicted.

Accurate masses of GST-GS16F1, GST-GS16F4, and GST-GS16F8 were, therefore, determined by MS (Figure 5a–c), and were found to be 37.7, 50.0, and 65.7 kDa for GST-GS16F1, GST-GS16F4, and GST-GS16F8, respectively, consistent with the predicted values of 37.4, 49.4, and 65.5 kDa. These results confirmed the successful expression and purification of all target proteins.

### 3.4. Analysis of Expression Levels

**Figure 6:**
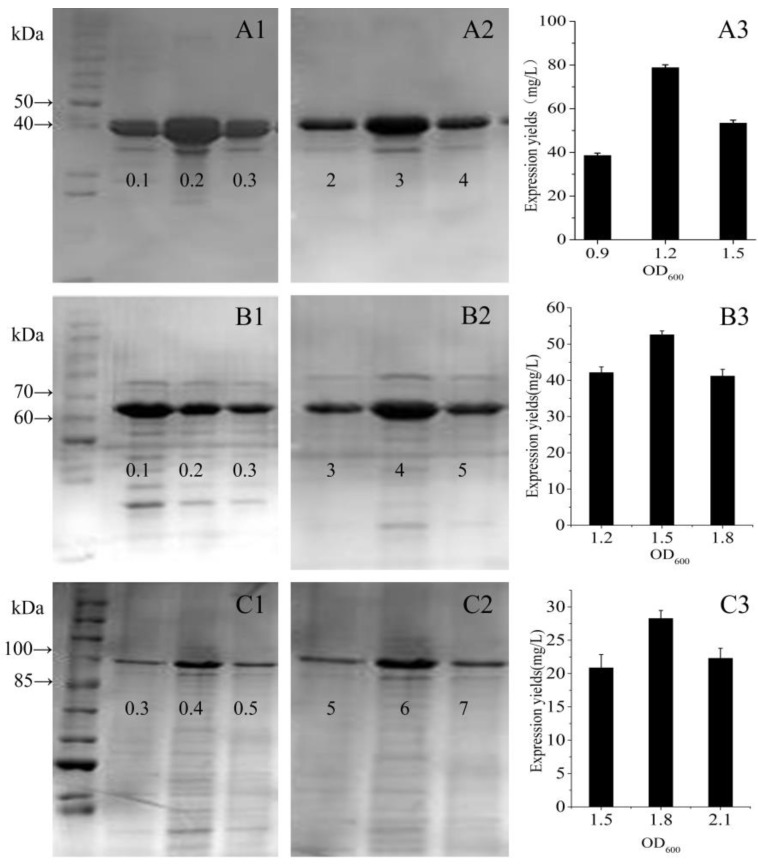
Expression level of each fusion protein by the three points method. (**A1**–**A3)** GST-GS16F1; (**B1**–**B3**) GST-GS16F4; and (**C1**–**C3**) GST-GS16F8. **A1**–**C1**: different IPTG concentration (mM); **A2**–**C2**: different induce time (h); and **A3**–**C3**: different initial cell density.

Based on the qualitative results of SDS-PAGE, we used a three-point design method to verify the optimum expression conditions and quantitatively determined the expression yields of the fusion proteins. For GST-GS16F1, with the optimal initial cell density and an induction time, the expression yield with 0.2 mM IPTG was twice that achieved with 0.1 mM or 0.3 mM IPTG (Figure 6A1). At the optimal initial cell density and with 0.2 mM IPTG, the expression yield after 3 h was significantly higher than that after 2 h or 4 h (Figure 6A2). With induction for 3 h with 0.2 mM IPTG, an expression yield of 79 mg per L of bacterial cells was achieved with an initial cell density (OD_600_) of 1.2 AU, compared with only 39 mg at an OD_600_ of 0.9 AU, and 53 mg at an OD_600_ of 1.5 AU (Figure 6A3). For GST-GS16F4, with an optimal initial cell density and induction time, the expression yield was highest with 0.1 mM IPTG, then decreased linearly with increasing IPTG concentration (Figure 6B1). With an optimal initial cell density and 0.1 mM IPTG, the expression yield after a 4 h induction was slightly higher than with a 3 h or 5 h induction (Figure 6B2). With induction for 4 h with 0.1 mM IPTG, an expression yield of 53 mg per L of bacterial cells was achieved with an initial cell density of 1.5 AU, which was significantly higher than with an OD_600_ of 1.2 AU (~42 mg) or 1.8 AU (~41 mg; Figure 6B3). For GST-GS16F8, with an optimal initial cell density and induction period, the expression yield was highest with 0.4 mM IPTG, but there was no significant difference among the other three IPTG concentrations tested (Figure 6C1). With optimal initial cell density and 0.4 mM IPTG, the expression yield was higher after a 6 h induction than a 5 h or 7 h induction (Figure 6C2). With induction for 6 h with 0.4 mM IPTG, an expression yield of ~28 mg per L bacterial cells was achieved with an initial cell density of 1.8 AU, compared with 21 mg for an OD_600_ of 1.5 AU and 22 mg for an OD_600_ of 2.1 AU (Figure 6C3). Furthermore, the expression yield generally decreased with increasing fusion protein molecular weight, and was highest for GST-GS16F1 and lowest for GST-GS16F8.

### 3.5. Amino Acid Analysis of GST-GS16Fn Proteins

The three fusion proteins GST-GS16F1, GST-GS16F4, and GST-GS16F8 include 373, 505, and 681 amino acid residues, respectively, mainly comprising Gly, Ala, Ser, Leu, Lys, Glu, Asp, Phe, and Pro. In all cases, the molecular weights closely resembled the predicted values and indicated the correct polypeptide expression and purification. As shown in Table 1, GST-GS16F1 primarily consists of Gly, Ala, Ser, Leu, Lys, Glu, Asp, and Pro, which constitute 75.4% (75.34% of the theoretical value) of all residues. GST-GS16F4 primarily consists of Gly, Ser, Ala, Glu, Leu, Asp, Phe, Pro, and Lys, which constitute 79.32% (83.21% of the theoretical value) of all residues, and GST-GS16F8 is primarily composed of Gly, Ser, Ala, Glu, Phe, Asp, Pro, and Leu, which constitute 83.61% (82.94% of the theoretical value) of all residues. The percentage deviated by no more than 4.67% from the predicted values in all cases, and the tested percentages of other amino acids were close to the predicted values. These results could indirectly suggest no gene mutations or substitutions occurred during DNA manipulation, transcription, or translation. Additionally, HCl acidolysis appeared to convert all Asn and Gln residues into Asp and Glu, since Asn and Gln were not detected and the percentages of Asp and Glu equaled the sum of both Asn + Asp and Gln + Glu.

### 3.6. Charge Analysis of GST-GS16Fn

**Figure 7:**
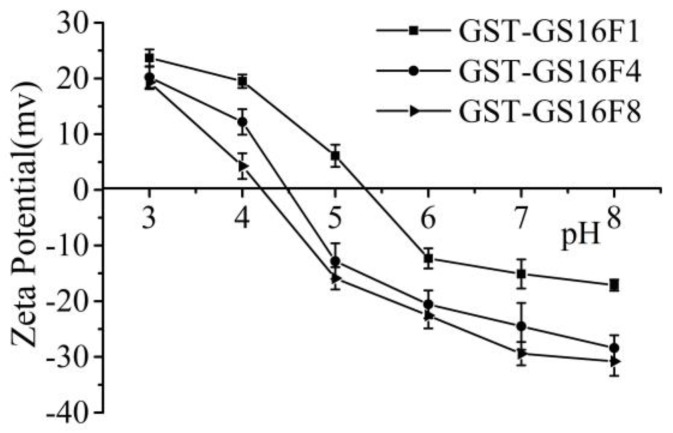
Zeta-potential in different pH environments of fusion proteins.

In the repetitive region (GS16) of the silk fibroin heavy chain, all three major amino acids (Gly, Ala and Ser) are neutral while, in the non-repetitive region (Fn), the number of Glu and Asp residues is greater than the number of basic residues, such as Lys. Specifically, the molar ratios are 6:1, 24:1, and 48:1 acidic vs. basic residues in F1, F4, and F8, respectively, suggesting these polypeptides are highly negatively charged. The number of acidic amino acids in the GST sequence is similar to the number of basic residues. As shown in Figure 7, all fusion proteins exhibited a negative *ζ*-potential in neutral aqueous solution, and the tested pI values for GST-GS16F1, GST-GS16F4 and GST-GS16F8 were 5.35, 4.5, and 4.2, respectively, consistent with the predicted values of 5.34, 4.44, and 4.09.

As negative charges (i.e., acidic polypeptides) tend to bind SDS difficultly, leading to the slow migration through the SDS-PAGE gel matrix. Some of the negative charge was offset with the presence of GST-tag, while electronegativity increased with the increase of the polymerization degree of the non-repetitive region (Fn). The result provided the reason why the GST-GS16F1 molecular weight was close to the theoretical value, while GST-GS16F4 and GST-GS16F8 appeared larger than theoretical, according to SDS-PAGE.

### 3.7. CD Analysis of Released Polypeptides GS16Fn

**Figure 8:**
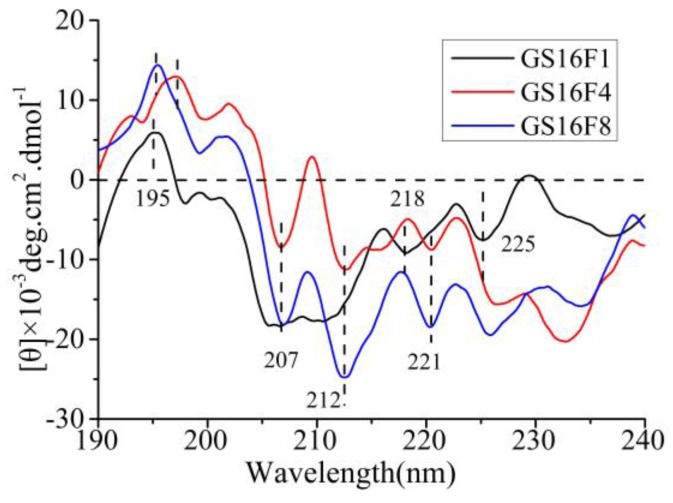
CD spectra of released polypeptides.

As shown in Figure 8, the CD spectra of GS16F1 exhibited a strong positive cotton effect peak at 195 nm, negative cotton effect peaks at 218 nm (β-sheet) and 225 nm (β-turn), and a wide weak negative peak around 207 nm (α-helix). The increase of the copy number of non-repetitive region (from GS16F1 to GS16F8) resulted in an obvious change of the molecular conformation. The negative cotton effect peak at 218 nm (β-sheet) disappeared, while the strong negative peaks of the *α*-helix structure at 207, 212, and 221 nm appeared. The molecular conformation of the polypeptides would be dynamically changed under different temperatures, concentrations, pH, and ions related to the preparation of biomaterials, which is the major investigation in our next work.

## 4. Conclusions

The *gs16f1* gene encoding a structural component of H-Chain was cloned, and recombinant *gs16f1*, *gs16f4*, and *gs16f8* containing different numbers of the components were stably expressed as GST fusion proteins in *E. coli*. The fusion proteins GST-GS16f1, GST-GS16F4, and GST-GS16F8 were successfully expressed and purified, as identified by SDS-PAGE, amino acid composition analysis, and mass spectrometry. In these recombinant polypeptides, the increase in the copy number of non-repetitive regions could lead to the changes of the molecular conformation from *β*-sheet (GS16F1) to *α*-helix (GS16F8). This work describes a method for the production of these polypeptides, and facilitates the investigation of their effect on the crystalline domains and amorphous regions of the H-Chain, as well their biological functions. We are focusing on investigating the dynamic transition of their molecular conformations under different conditions (temperature, concentration, pH, ions, etc.) related to the preparation of biomaterials, and their effects on cellular responses.

## Figures and Tables

**Table 1:** Amino acid composition of three fusion proteins.

Amino Acid	GST-GS16F1 (mol%)		GST-GS16F4 (mol%)		GST-GS16F8 (mol%)	
	Theoretical	Experimental	Theoretical	Experimental	Theoretical	Experimental
Asp	5.60	5.64	5.38	5.45	5.22	5.11
Thr	1.96	1.96	1.45	1.42	1.08	0.99
Ser	11.20	10.97	15.73	15.48	19.05	19.35
Glu	5.60	5.62	6.63	6.32	7.37	7.48
Gly	20.73	20.56	21.53	20.17	22.12	22.73
Ala	13.45	13.62	13.66	12.73	13.82	13.96
Cys	1.12	1.17	0.83	7.09	0.61	1.60
Val	3.36	3.25	3.73	3.55	3.99	4.02
Met	2.52	2.69	1.86	0.00	1.38	0.00
Ile	3.64	3.72	2.69	2.47	2.00	1.99
Leu	8.12	8.20	6.00	5.75	4.45	4.29
Tyr	3.92	3.87	2.90	2.88	2.15	2.23
Phe	3.64	3.57	5.18	5.08	6.30	6.46
Lys	6.16	6.29	4.55	4.00	3.38	3.33
His	1.68	1.73	1.24	1.18	0.92	0.92
Arg	2.80	2.56	2.07	1.90	1.54	1.74
Pro	4.48	4.50	4.55	4.34	4.61	4.23
Total	≈100		≈100		≈100	

## Abbreviations

IPTG	isopropyl-β-d-thiogalactoside
SDS	sodium dodecyl sulphate
PAGE	polyacrylamide gel electrophoresis
GST	glutathione S-transferase
gst	genes encoding GST
GST-GS16F1	recombinant GST and GS16F1 fusion protein
GST-GS16F4	recombinant GST and GS16F4 fusion protein
GST-GS16F8	recombinant GST and GS16F8 fusion protein
pI	isoelectric point
*E. coli*	*Escherichia coli*

### Amino Acids

G	Gly
S	Ser
V	Val
A	Ala
P	Pro
F	Phe
N	Asn
E	Glu
D	Asp
